# *Pseudomonas aeruginosa* prioritizes detoxification of hydrogen peroxide over nitric oxide

**DOI:** 10.1186/s13104-021-05534-7

**Published:** 2021-03-26

**Authors:** Darshan M. Sivaloganathan, Mark P. Brynildsen

**Affiliations:** 1 Program in Quantitative and Computational Biology, Princeton University, Princeton, NJ USA; 2 Department of Chemical and Biological Engineering, Princeton University, Princeton, NJ USA

**Keywords:** Fhp, Catalase, Hydroperoxide reductase, NO, H_2_O_2_, Antimicrobial, Phagosome

## Abstract

**Objective:**

Bacteria are exposed to multiple concurrent antimicrobial stressors within phagosomes. Among the antimicrobials produced, hydrogen peroxide and nitric oxide are two of the most deleterious products. In a previous study, we discovered that when faced with both stressors simultaneously, *Escherichia coli* prioritized detoxification of hydrogen peroxide over nitric oxide. In this study, we investigated whether such a process was conserved in another bacterium, *Pseudomonas aeruginosa.*

**Results:**

*P. aeruginosa* prioritized hydrogen peroxide detoxification in a dose-dependent manner. Specifically, hydrogen peroxide detoxification was unperturbed by the presence of nitric oxide, whereas larger doses of hydrogen peroxide produced longer delays in nitric oxide detoxification. Computational modelling revealed that the rate of nitric oxide consumption in co-treated cultures was biphasic, with cells entering the second phase of detoxification only after hydrogen peroxide was eliminated from the culture.

**Supplementary Information:**

The online version contains supplementary material available at 10.1186/s13104-021-05534-7.

## Introduction

Phagosomes are complex environments in which bacteria are exposed concurrently to a multitude of stressors [1–3]. Among these antimicrobials are nitric oxide (NO) and hydrogen peroxide (H_2_O_2_) [1, 3, 4]. Both NO and H_2_O_2_ rapidly diffuse across bacterial membranes and are capable of damaging a diverse array of biomolecules within cells [5–9]. NO can directly damage iron-sulfur clusters in proteins and block cellular respiration by reversibly binding heme groups [6, 7]. Moreover, NO can react with oxygen and superoxide to produce even more toxic molecules, termed reactive nitrogen species (RNS), that can cause lipid peroxidation, DNA deamination, and nitrosylation of thiols and tyrosines [6, 9]. Similarly, H_2_O_2_ can damage proteins by disrupting iron-sulfur clusters and reacting with specific amino acid residues, such as cysteine and methionine [5, 8]. Further, H_2_O_2_ can react with ferrous iron to generate hydroxyl radical, which is an even more deleterious species that is capable of reacting with a wide array of biomolecules within cells, including nucleic acids, lipids, sugars, and amino acids [8, 10].

Bacteria have evolved detoxification systems to combat these stressors. For example, *Escherichia coli* possess an NO dioxygenase (Hmp) and an NO reductase (NorV) to eliminate NO under aerobic and anaerobic conditions, respectively [6, 9]. To detoxify H_2_O_2_, *E. coli* has one alkyl hydroperoxide reductase (Ahp) and two catalases (KatE and KatG) [5]. While much has been uncovered regarding how bacteria, such as *E. coli*, respond to NO and H_2_O_2_ treatment separately, less is known about how microbes respond to concurrent treatment. In a previous study, we investigated the response of *E. coli* to concurrent treatment with NO and H_2_O_2_ at concentrations reflective of phagosomal compartments (μM) [11]. We observed that *E. coli* prioritizes H_2_O_2_ elimination over NO in a dose-dependent manner. Specifically, NO detoxification was significantly impaired by H_2_O_2_ (with larger doses corresponding to greater impairment), whereas H_2_O_2_ detoxification was unperturbed by NO at the concentrations investigated. A deeper analysis revealed that increasing doses of H_2_O_2_ impaired both transcription and translation of the major NO detoxification protein, Hmp, under aerobic conditions. Such a phenomenon has noticeable parallels with carbon catabolite repression (CCR), which occurs in environments with multiple carbon sources when microbes consume specific nutrients prior to others [12]. CCR has been widely observed across many bacterial species, with the preferred consumption of glucose over lactose by *E. coli* providing the prototypical example [12].

In this study, we were interested in exploring whether the prioritization of H_2_O_2_ over NO, which we previously observed in *E. coli*, was conserved across different bacterial species. In particular, we investigated dual stress conditions in *Pseudomonas aeruginosa*, which differs significantly from *E. coli* despite both being Gram-negative bacteria. *P. aeruginosa* inhabits very different niches in the human body, such as the airways and skin, compared to *E. coli*, which thrives in the gastrointestinal system [13, 14]. Genetically, *P. aeruginosa* and *E. coli* can harbor significantly different-sized genomes (e.g.*,* ~ 6.3·10^6^ base pairs for *P. aeruginosa* PAO1, ~ 4.6·10^6^ base pairs for *E. coli* MG1655), whereas, metabolically, *P. aeruginosa* prefers a gluconeogenic metabolism (e.g.*,* preferential consumption of succinate over glucose) and *E. coli* prefers a glycolytic metabolism (e.g.*,* preferential consumption of glucose over succinate) [15–17]. Moreover, *P. aeruginosa* contains a different array of NO and H_2_O_2_ detoxification enzymes. Similar to *E. coli*, *P. aeruginosa* contains an NO dioxygenase (Fhp) and an NO reductase (NorCB), which are responsible for eliminating NO under aerobic and anaerobic conditions, respectively. However, NorCB uses a heme center for catalysis, whereas NorV uses a non-heme di-iron active site, and *P. aeruginosa* has a nitrite reductase (NirS) that generates NO, while *E. coli* does not [18–21]. With regard to H_2_O_2_, *P. aeruginosa* possesses three alkyl hydro-peroxidases (AhpB, AhpC, Ohr) and three catalases (KatA, KatB, KatE), whereas *E. coli* contains one alkyl hydroperoxidase (AhpCF) and two catalases (KatG, KatE) [22]. For these reasons, we examined whether a similar prioritized detoxification of H_2_O_2_ and NO would be observed with *P. aeruginosa*.

## Main text

### Materials and methods

#### Bacterial strains

All experiments were performed using *P. aeruginosa* PAO1 (ATCC 15692).

### Chemicals and growth media

All experiments were conducted in basal salts media (BSM) supplemented with 15 mM succinate. The NO donor, (Z)-1-[N-(3-aminopropyl)-N-(3-ammoniopropyl)amino]diazen-1-ium-1,2-diolate (DPTA NONOate), was dissolved in 10 mM NaOH and stored on ice prior to use. H_2_O_2_ solution used was 35 wt. % in water and was diluted to different stock concentrations (10 mM and 20 mM) in autoclaved Milli-Q water (18.2 MΩ cm at 25 °C). Luria–Bertani (LB) broth was made by dissolving LB powder in Milli-Q water and autoclaving the solution. Similarly, LB agar plates with pyruvate were made by dissolving LB powder and agar in Milli-Q water and autoclaving. After the solution had cooled, pyruvate was added at a concentration of 25 mM and the solution was poured into sterile petri dishes. Pyruvate was used to scavenge any residual H_2_O_2_ from samples once plated.

### [NO] and [H_2_O_2_] measurements

Continuous measurement of NO concentrations was achieved using a 2 mm nitric oxide sensing probe (World Precision Instruments). The sensor was calibrated daily by adding increasing doses of SNAP (S-Nitroso-N-Acetyl-D,L-Penicillamine) to 10 mL of 0.1 M CuCl_2_ solution per the manufacturer’s instructions. A conversion factor of 0.457 molecules of NO per molecule of SNAP was used to convert calibration data to units of NO concentration [23]. H_2_O_2_ concentrations were determined using Amplex Red hydrogen peroxide/peroxidase kits (Life Technologies), per the manufacturer’s instructions. Samples were diluted to less than 10 µM and a standard curve with known concentrations (0, 1, 2.5, 5 and 10 µM) was used to convert fluorescence values to H_2_O_2_ concentrations.

### [NO] and [H_2_O_2_] consumption assays

*P. aeruginosa* was taken from a − 80 °C frozen stock, inoculated into 1 mL of LB media, and grown for 16 h in an incubator at 37 °C and 250 revolutions per minute (rpm). After 16 h, the overnight culture was inoculated into 20 mL of BSM minimal media in a 250 mL baffled flask at an optical density at 600 nm (OD_600_) of 0.01. The flask was incubated at 37 °C and 250 rpm until cells reached mid-exponential phase (OD_600_ ~ 0.2). When the culture reached the desired OD_600_, 8 mL of culture was transferred to 8 microcentrifuge tubes and spun at 15,000 rpm for 3 min. After centrifugation, 980 µL of supernatant was removed from each tube and cells were concentrated into 1 mL of BSM media. Before inoculation of cells into the bioreactor, 10 µL of the appropriate stock solution of H_2_O_2_ was added to a bioreactor containing 10 mL of BSM media to reach a starting concentration of 10 or 20 µM. In assays performed in the absence of H_2_O_2_, 10 µL of autoclaved MilliQ water was added instead. Concentrated cell culture was added to bioreactors to achieve an initial OD_600_ of 0.025. Immediately after inoculation, 6.95 µL of 72 mM DPTA NONOate was added to obtain an initial concentration of 50 µM within the bioreactor, and the NO concentration was monitored continuously. In assays performed in the absence of NO, the appropriate volume of 10 mM NaOH was added instead. At each time point, 150 µL of solution was removed and sterile filtered using a 0.22 µM syringe filter (Millex) to provide samples for H_2_O_2_ measurements. Samples for initial time points (t = 0) were removed prior to inoculation with cells. For assays lacking H_2_O_2_, 150 µL was removed at each time point to maintain equivalent reactor volumes throughout the assay.

### Cell culturability measurements

To measure cell culturability, 200 µL of solution was removed at time points, transferred to microcentrifuge tubes, and spun at 15,000 rpm for 3 min. Afterwards, 180 µL of supernatant was removed, and the cell pellet was re-suspended in 980 µL of phosphate buffered saline (PBS). The samples were then serially diluted in PBS and plated on LB agar supplemented with 25 mM pyruvate. Plates were incubated at 37 °C for 16 h at which time colonies were counted.

### Mathematical modelling

The model used was constructed in previous studies [6, 21, 24–29]. For this study, however, the model was simplified and reduced to a system of only three ordinary differential equations to capture NO dynamics observed in a cell-free bioreactor upon delivery of 50 µM of the NO donor DPTA NONOate, where *k*_*NONOate*,_ *k*_*autox*,_ *k*_*La,NO*_ and *k*_*La,O2*_ are rate constants for NONOate degradation, NO autoxidation, NO mass transfer, and O_2_ mass transfer, respectively. [O_2_]_sat_ refers to the dissolved oxygen concentration in equilibrium with air, whereas [O_2_], [NO], and [DPTA] refer to the O_2_, NO, and DPTA NONOate concentrations within the bioreactor.

1 $$\begin{array}{*{20}c} {\frac{{d\left[ {NO} \right]}}{dt} = 2 \cdot k_{NONOate} \cdot \left[ {NONOate} \right] - 2 \cdot k_{autox} \left[ {NO} \right]^{2} \left[ {O_{2} } \right] - k_{La,NO} \cdot \left[ {NO} \right]} \\ \end{array}$$

2 $$\begin{array}{*{20}c} {\frac{{d\left[ {O_{2} } \right]}}{dt} = k_{La,O2} \cdot \left( {\left[ {O_{2} } \right]_{sat} - \left[ {O_{2} } \right]} \right) - k_{autox} \cdot \left[ {NO} \right]^{2} \left[ {O_{2} } \right]} \\ \end{array}$$

3 $$\begin{array}{*{20}c} {\frac{{d\left[ {NONOate} \right]}}{dt} = - k_{NONOate} \cdot \left[ {NONOate} \right]} \\ \end{array}$$

### Parameter fitting

Parameters were fit based on experimental data performed in a cell-free bioreactor dosed with 50 µM DPTA NONOate. Specifically, the initial concentration of NO was set to zero, DPTA NONOate was set to 50 µM, and both [O_2_]_sat_ and the initial [O_2_] were set to 210 µM. The value for *k*_*La,O2*_ was obtained from a previous study using an identical apparatus [21]. The remaining parameters (*k*_*NONOate*,_ *k*_*autox*,_ *k*_*La,NO*_) were optimized using a non-linear least squares regression algorithm (lsqcurvefit) that minimized the sum of the squared residual errors (SSR) between measured data and simulation data. One hundred initializations were performed using randomized initial values within previously established bounds [27]. Evidence ratios (ER) were calculated, and all parameters sets with an ER less than 10 were accepted as viable. Sixty-eight parameters sets were retained and a comparison between measured data and simulations performed with the optimal set (ER = 1) is plotted in Additional file 1: Figure S1A.

### Black-box modelling

Due to the large size of the ensemble and the tight clustering of viable parameter sets (Additional file 1: Figure S1B), only the optimal parameter set was used to estimate cellular consumption using a black-box model. At each time interval in the experimental data, simulations were performed to estimate the rate of NO generated by DPTA NONOate, the rate of NO loss by autoxidation, and mass transport of NO to the gas phase. These values were used to calculate the change in NO in that time interval that could be attributed to abiotic means, which was subtracted from d[NO]/dt from the experimental data to calculate the rate of NO consumption by cells. The procedure was carried out at all time points up to NO clearance, defined as [NO] less than or equal to 0.2 µM, and the cumulative consumption of NO over time was calculated. NO consumption rates were estimated for each condition by fitting linear portions of curves with lines of best fit and computing the slopes (Additional file 2: Figure S2). The number of points to include when estimating the line of best fit was chosen based on the maximum number of points in which the SSR did not dramatically increase between consumption curves and the best-fit line.

## Results

**Fig. 1:**
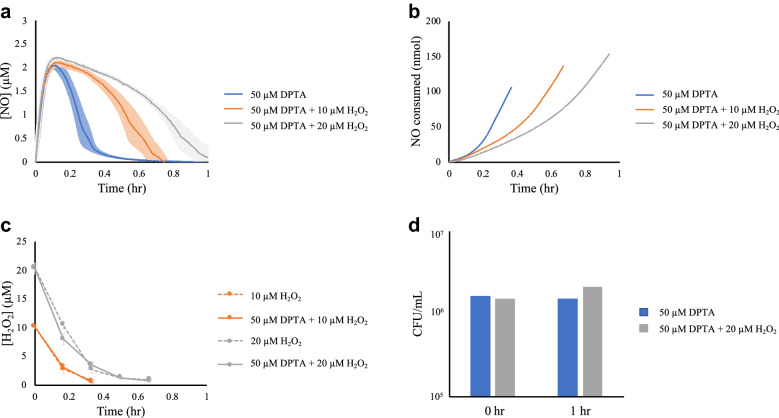
H_2_O_2_ clearance is prioritized over that of NO. *P. aeruginosa* cultures were grown to exponential phase and inoculated, at an OD_600_ of 0.025, into a bioreactor containing either 0, 10, or 20 μM H_2_O_2_. Immediately after addition of cells, cultures were treated with either 50 μM DPTA NONOate or the same volume of the DPTA NONOate solvent. **a** NO concentrations in the bioreactor were continuously measured. **b** Cumulative cellular NO consumption was assessed using a kinetic model with a black-box cellular compartment. **c** H_2_O_2_ concentrations were measured at 10-min intervals. **d** Culturability of *P. aeruginosa* in the presence of 50 μM DPTA and 50 μM DPTA + 20 μM H_2_O_2_ were assessed at the beginning and 1 h after treatment. All data represents the mean of three replicates, with error bars representing the standard error of the mean

In this study, we explored the relationship between H_2_O_2_ and NO detoxification in *P. aeruginosa*. Experimental conditions were chosen to mirror our previous study on *E. coli* [11]*.* Specifically, *P. aeruginosa* cells were grown to exponential phase and introduced into a bioreactor at an OD_600_ of 0.025. Immediately after addition of cells, an NO donor (DPTA NONOate) was added at a concentration of 50 μM to the reactor, as well as different concentrations of H_2_O_2_ (0, 10, or 20 μM). Increasing concentrations of H_2_O_2_ delayed NO detoxification by cells in a dose-dependent fashion (Fig. 1a). Similar to what was observed in *E. coli*, NO was detoxified in a biphasic manner (Fig. 1b). Initial NO consumption rates were similar across all treatment conditions (~ 100 nmol per hour). The second phase of consumption rates deviated somewhat across culture conditions, but were all over threefold higher than initial rates. The drastic increases in consumption rates were only observed after detoxification of H_2_O_2._ Moreover, H_2_O_2_ clearance was unaffected by the presence of NO (Fig. 1c). Further, the culturability of samples exposed to NO and combination treatments of NO and H_2_O_2_ were comparable (Fig. 1d). Overall, the data demonstrated that *P. aeruginosa* also prioritized detoxification of H_2_O_2_ over the detoxification of NO.

## Discussion

Numerous bacteria have defense systems for immune antimicrobials that help them propagate infections [30–34]. Among those antimicrobials are NO and H_2_O_2_, which are capable of inducing widespread cytotoxic effects on phagocytized bacteria [7, 8]. In a previous study, we investigated how *E. coli* responds to simultaneous NO and H_2_O_2_ exposure, and discovered that it prioritizes H_2_O_2_ elimination over that of NO [11]. Further, we found that the phenomenon was regulated at both the transcriptional and translational levels, which was reminiscent of CCR [11, 12]. In this study, we investigated whether prioritized detoxification translated to *P. aeruginosa*. Interestingly, we observed that, similar to *E. coli, P. aeruginosa* NO detoxification was significantly delayed by cotreatment with H_2_O_2_, whereas H_2_O_2_ detoxification was unimpeded by NO. Those results demonstrated that prioritized detoxification of these antimicrobials is not unique to *E. coli* and extends to other bacteria. Such a phenomenon may represent a highly conserved defensive strategy that bacteria use in multi-stress conditions, much like they use CCR in multi-nutrient conditions [12]. Looking forward, understanding the mechanistic bases of prioritized detoxification could lead to strategies to treat bacteria that use NO and H_2_O_2_ detoxification systems to enhance their virulence [9]. Such an anti-infective approach is currently being explored [35], along with other alternative treatments [36–40], with the ultimate goal of complementing currently available antibiotics.

## Limitations

Further investigation into potential mechanisms for the prioritized detoxification in *P. aeruginosa* has not been performed. An assessment of Fhp transcription, translation, and catalytic activity under both NO and NO with H_2_O_2_ stress conditions will need to be evaluated.

## Supplementary Information

**Additional file 1: Figure S1.** Training of extracellular parameters. (A) Fifty μM DPTA NONOate was added to a cell-free bioreactor and [NO] was continuously measured (blue line). The measured data is the mean of three replicates, with error bars representing the standard error of the mean. The data was used to train parameters in a kinetic model of NO reactivity and transport in the absence of cells. All parameter sets with ER < 10 were retained and considered viable sets. Due to the size of the ensemble and the tight clustering of parameter sets, simulations are plotted for only the optimal parameter set (minimum SSR, ER = 1) (orange line). (B) A table containing the optimal, minimum, and maximum parameter values within the ensemble.

**Additional file 2: Figure S2.** Biphasic NO consumption rates under different treatment conditions. (A) 50 μM DPTA. (B) 50 μM DPTA + 10 μM DPTA. (C) 50 μM DPTA + 20 μM DPTA. For each condition the rate of NO consumption for each regime was approximated by calculating the slope of the line of best fit. The equations of each line of best fit, and R^2^ value are provided.

## Data Availability

The datasets generated for this study are available on request to the corresponding author.

## Abbreviations

NO: Nitric oxide
H_2_O_2_: Hydrogen peroxide
RNS: Reactive nitrogen species
CCR: Carbon catabolite repression
BSM: Basal salts media
DPTA NONOate: (Z)-1-[N-(3-aminopropyl)-N-(3-ammoniopropyl)amino]diazen-1-ium-1,2-diolate
LB: Luria–Bertani
SNAP: S-Nitroso-N-Acetyl-D,L-Penicillamine
RPM: Revolutions per minute
OD_600_: Optical density at 600 nm
PBS: Phosphate buffered saline
SSR: Sum of square residuals
ER: Evidence ratios
