# Correction to: *Pseudomonas aeruginosa* prioritizes detoxification of hydrogen peroxide over nitric oxide

**DOI:** 10.1186/s13104-021-05669-7

**Published:** 2021-07-12

**Authors:** Darshan M. Sivaloganathan, Mark P. Brynildsen

**Affiliations:** 1 Program in Quantitative and Computational Biology, Princeton University, Princeton, NJ USA; 2 Department of Chemical and Biological Engineering, Princeton University, Princeton, NJ USA

## Correction to: BMC Res Notes (2021) 14:120 https://doi.org/10.1186/s13104-021-05534-7

Following the publication of the original article [1], the authors brought to our attention that an error was introduced in Figure 1 during the implementation of their corrections: The error bars that were present in panel d of Figure 1 were accidentally removed.

**Fig. 1:**
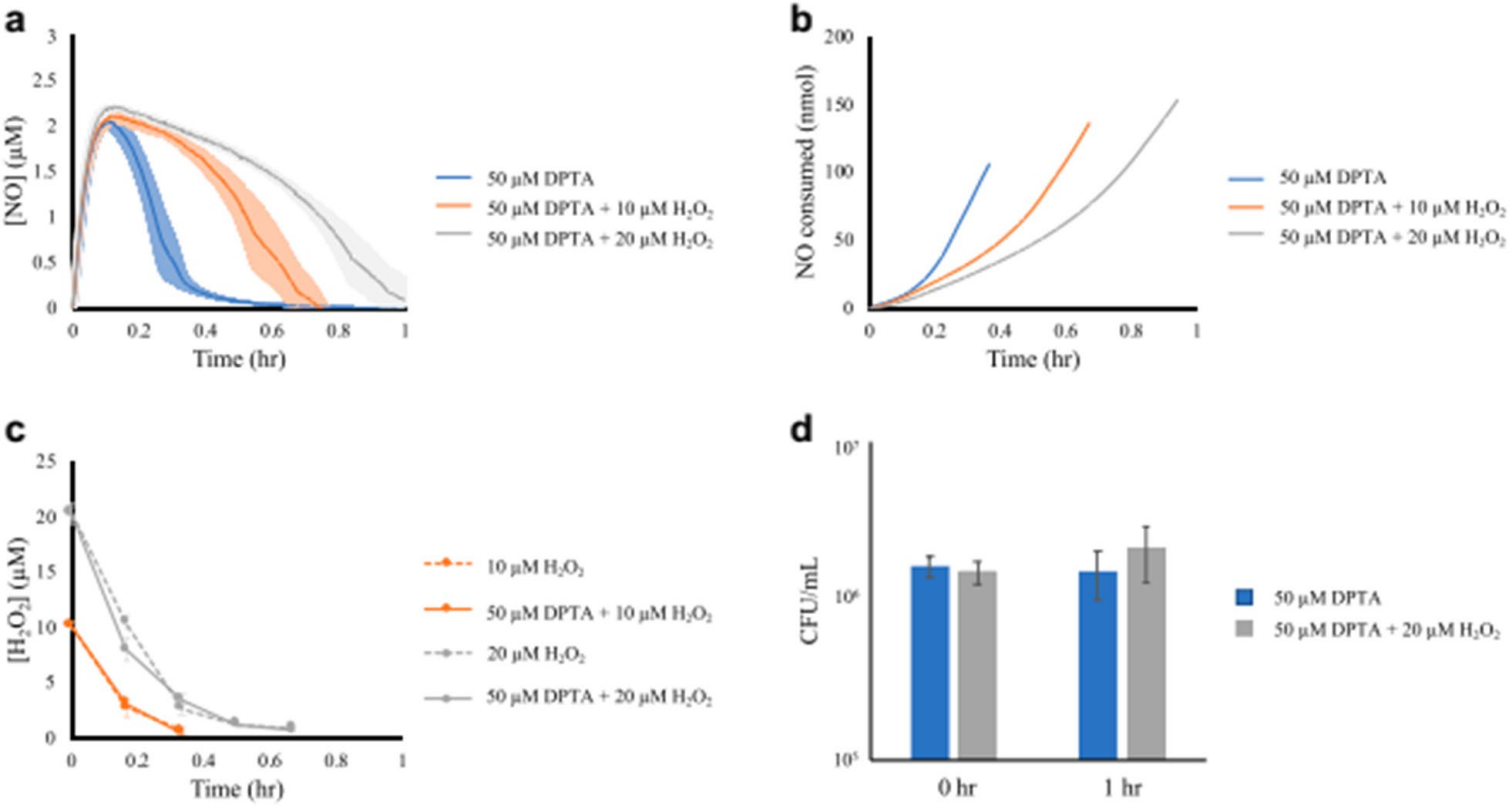
H_2_O_2_ clearance is prioritized over that of NO. *P. aeruginosa* cultures were grown to exponential phase and inoculated, at an OD_600_ of 0.025, into a bioreactor containing either 0, 10, or 20 μM H_2_O_2_. Immediately after addition of cells, cultures were treated with either 50 μM DPTA NONOate or the same volume of the DPTA NONOate solvent. **a** NO concentrations in the bioreactor were continuously measured. **b** Cumulative cellular NO consumption was assessed using a kinetic model with a black-box cellular compartment. **c** H_2_O_2_ concentrations were measured at 10-min intervals. **d** Culturability of *P. aeruginosa* in the presence of 50 μM DPTA and 50 μM DPTA + 20 μM H_2_O_2_ were assessed at the beginning and 1 h after treatment. All data represents the mean of three replicates, with error bars representing the standard error of the mean

The correct Figure 1 is shown here below and has now been updated in the original article.
