# Supplementary figures and images for: Pseudomonas aeruginosa prioritizes detoxification of hydrogen peroxide over nitric oxide
Source: BMC Res Notes. 2021 Mar 26;14:120. doi: 10.1186/s13104-021-05534-7 (PMC7995768; doi:10.1186/s13104-021-05534-7)

A

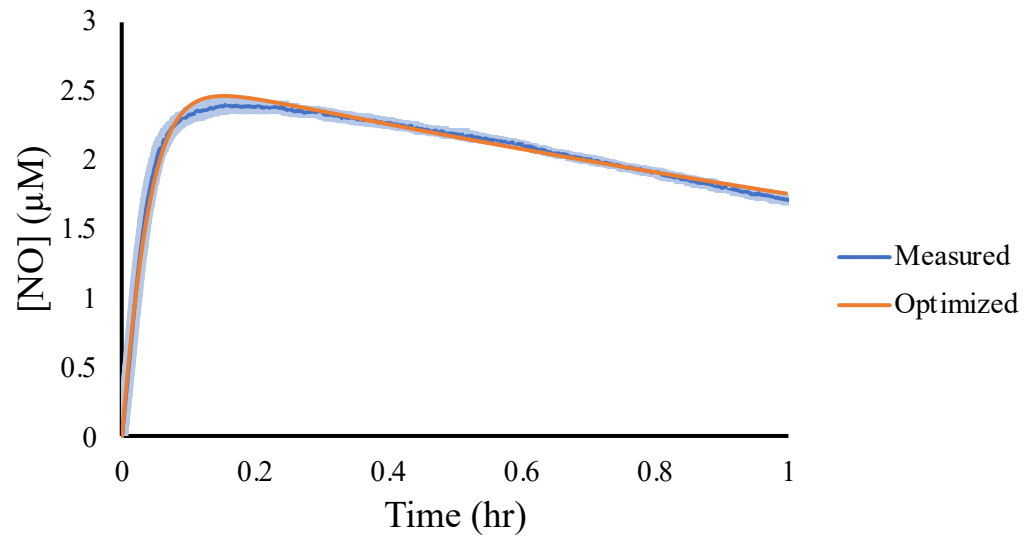

B

| Parameter     | Optimal  | Min      | Max      | Units                           |
|---------------|----------|----------|----------|---------------------------------|
| $k_{autox}$   | 0.00429  | 0.00426  | 0.00432  | $\mu\text{M}^{-2}\text{h}^{-1}$ |
| $k_{NONOate}$ | 0.57787  | 0.57707  | 0.57822  | $\text{h}^{-1}$                 |
| $k_{LaNO}$    | 12.54234 | 12.48849 | 12.57517 | $\text{h}^{-1}$                 |

Supplement: Supplementary file 1 — Additional file 1: Figure S1. Training of extracellular parameters. (A) Fifty μM DPTA NONOate was added to a cell-free bioreactor and [NO] was continuously measured (blue line). The measured data is the mean of three replicates, with error bars representing the standard error of the mean. The data was used to train parameters in a kinetic model of NO reactivity and transport in the absence of cells. All parameter sets with ER < 10 were retained and considered viable sets. Due to the size of the ensemble and the tight clustering of parameter sets, simulations are plotted for only the optimal parameter set (minimum SSR, ER = 1) (orange line). (B) A table containing the optimal, minimum, and maximum parameter values within the ensemble. [file 13104_2021_5534_MOESM1_ESM.pdf]

A

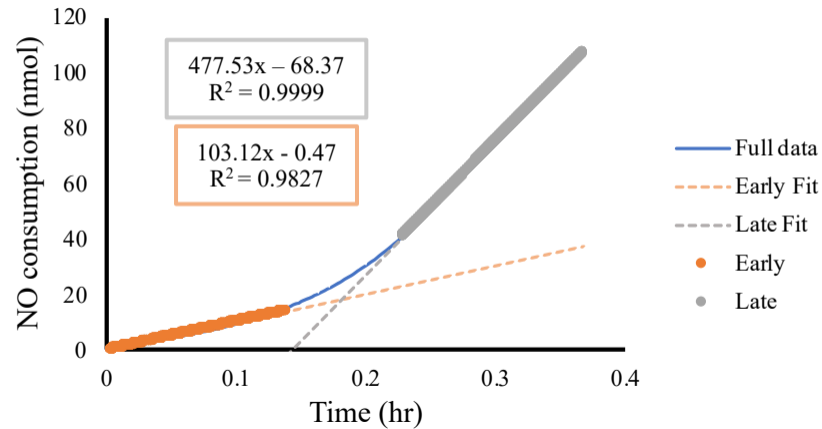

B

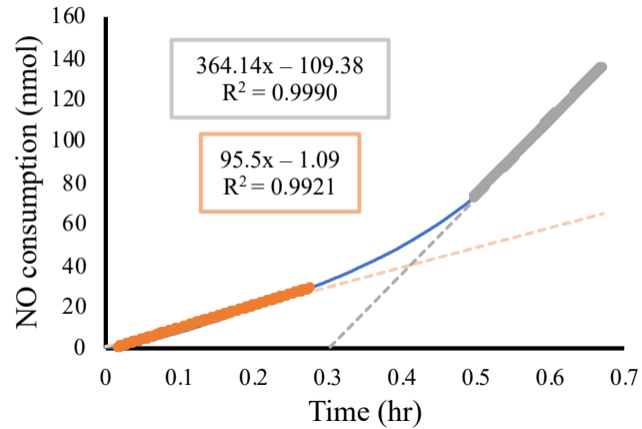

C

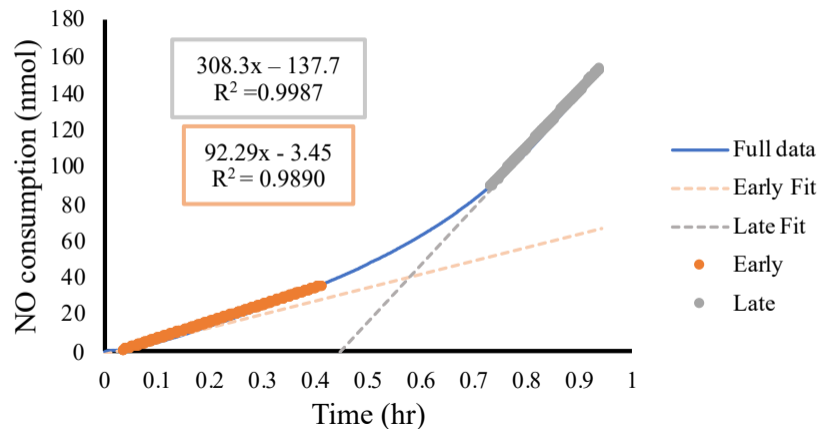

Supplement: Supplementary file 2 — Additional file 2: Figure S2. Biphasic NO consumption rates under different treatment conditions. (A) 50 μM DPTA. (B) 50 μM DPTA + 10 μM DPTA. (C) 50 μM DPTA + 20 μM DPTA. For each condition the rate of NO consumption for each regime was approximated by calculating the slope of the line of best fit. The equations of each line of best fit, and R2 value are provided. [file 13104_2021_5534_MOESM2_ESM.pdf]
